# Characteristics of Patients for Whom Benznidazole Was Released Through the CDC-Sponsored Investigational New Drug Program for Treatment of Chagas Disease — United States, 2011–2018

**DOI:** 10.15585/mmwr.mm6729a3

**Published:** 2018-07-27

**Authors:** Barbara L. Herwaldt, Cindy P. Dougherty, Christopher K. Allen, Julian P. Jolly, Megan N. Brown, Patricia Yu, Yon Yu

**Affiliations:** ^1^Parasitic Diseases Branch, Division of Parasitic Diseases and Malaria, Center for Global Health, CDC; ^2^CDC Drug Service, Division of Scientific Resources, National Center for Emerging and Zoonotic Infectious Diseases, CDC; ^3^Regulatory Affairs, Office of the Director, National Center for Emerging and Zoonotic Infectious Diseases, CDC.

Chagas disease (also known as American trypanosomiasis) is caused by the protozoan parasite *Trypanosoma cruzi* (*1*,*2*). Vectorborne transmission via skin or mucosal contact with the feces of infected triatomine bugs mainly occurs in rural areas of Latin America but has been reported in the southern United States (*3*). The parasite also is transmissible congenitally and via blood transfusion, organ transplantation, and accidental laboratory exposures. The two drugs used for treating Chagas disease are benznidazole and nifurtimox (*1*,*2*), which have been used in Latin America since the 1970s and 1960s, respectively. In the absence of commercially available drugs approved by the Food and Drug Administration (FDA), benznidazole and nifurtimox have been available exclusively through CDC, under Investigational New Drug (IND) treatment protocols. On August 29, 2017, FDA approved a benznidazole product (Chemo Research, SL, in care of Exeltis*) for treatment of Chagas disease (*4*), which became commercially available on May 14, 2018. Therefore, effective May 14, 2018, benznidazole is no longer available through the CDC-sponsored IND program. This report summarizes selected characteristics of patients for whom CDC released benznidazole through that program from October 2011, when the IND went into effect, until mid-May 2018. The majority of the 365 patients included in intention-to-treat analyses were chronically infected adults who were born and became infected in Latin America. Physician requests for benznidazole should now be directed to the drug company Exeltis.^†^ The CDC-sponsored IND for nifurtimox remains in effect to provide an alternative therapeutic option to benznidazole when clinically appropriate. CDC will continue to provide reference diagnostic testing for *T. cruzi* infection and teleconsultative services regarding Chagas disease.

## Background

*Trypanosoma cruzi* infection occurs in two successive phases. The acute phase, which can be life-threatening in immunosuppressed persons, typically lasts for several weeks or months. The subsequent chronic phase, which can be life-threatening (e.g., can cause sudden cardiac death) even in asymptomatic persons, is associated with an estimated 20%–30% lifetime risk of developing cardiac or gastrointestinal disease (*1*,*2*). The number of chronically infected Latin American immigrants in the United States has been estimated to exceed 300,000 (*5*,*6*). Blood-donor screening for serologic evidence of *T. cruzi* infection, which was introduced in the United States in 2007, has resulted in increased detection of asymptomatic, chronically infected persons and has helped raise awareness about Chagas disease, including the importance of diagnostic testing and appropriate antimicrobial therapy for infected persons.

## Treatment of Chagas Disease in U.S. Patients

To ensure availability of and access to antimicrobial therapy for eligible U.S. patients, CDC has sponsored expanded-access IND programs for benznidazole (IND 103,359) and nifurtimox (IND 84,422). Both drugs are administered orally, typically for approximately 2 months (benznidazole) or approximately 3–4 months (nifurtimox). Both drugs are commonly associated with adverse events, which tend to be more frequent and bothersome in adults than in children (*1*,*2*). Some patients tolerate one drug better than the other; if one of the drugs is not tolerated, the other can be tried as an alternative.

The CDC-sponsored IND for benznidazole went into effect in October 2011. The IND treatment program used benznidazole manufactured by a Brazilian public pharmaceutical laboratory, Laboratório Farmacêutico do Estado de Pernambuco (LAFEPE), which was the sole producer of benznidazole when the protocol went into effect.

On August 29, 2017, FDA approved benznidazole (Chemo Research, SL, in care of Exeltis) for treatment of Chagas disease in children aged 2–12 years^§^ (*4*). On May 14, 2018, the FDA-approved benznidazole product became commercially available through an exclusive distributor, Foundation Care. Physician requests for benznidazole for treatment of Chagas disease in U.S. patients (not limited to patients aged 2–12 years) (*7*) should now be directed to Exeltis.

Effective May 14, 2018, benznidazole is no longer available through the CDC-sponsored IND program. However, the IND will remain in effect through November 2018 to provide sufficient time for patients who were enrolled before the FDA-approved product became commercially available to complete their current treatment course and for their physicians to comply with IND reporting requirements. The CDC-sponsored IND for nifurtimox remains in effect to provide an alternative therapeutic option to benznidazole when clinically appropriate (e.g., for patients who do not tolerate benznidazole therapy).

## Characteristics of Patients for Whom Benznidazole Was Released Through the CDC-Sponsored IND Program

From October 2011 until patient enrollment was discontinued in May 2018, CDC released the LAFEPE benznidazole product under the IND for 369 patients, including two patients who received benznidazole prophylaxis after laboratory accidents and two patients determined not to have Chagas disease after benznidazole was released. Data for the remaining 365 patients were included in intention-to-treat analyses. Median patient age was 42.9 years (range = 0.1–76.0 years). Only 32 patients (8.8%) were aged <19 years (Table), including two patients (0.5%) aged 2–12 years (5 years and 9 years) and one neonate with congenital Chagas disease (*8*).

**TABLE:** Number (N = 365*) and percentage of patients for whom benznidazole was released through the CDC-sponsored Investigational New Drug program for treatment of Chagas disease, by selected characteristics — United States, October 2011–May 2018^†^

Characteristic	No.	(%)
**Sex**		
Female	192	(52.6)
Male	173	(47.4)
**Age group (yrs)**		
<2	1	(0.3)
2–12	2	(0.5)
13–18	29	(7.9)
19–50	236	(64.7)
>50	97	(26.6)
**Country of birth in Latin America^§^**		
**Total**	**319**	**(87.4)**
El Salvador	117	(32.1)
Mexico	77	(21.1)
Bolivia	67	(18.4)
Guatemala	16	(4.4)
Honduras	14	(3.8)
Brazil	8	(2.2)
Colombia	5	(1.4)
Argentina	4	(1.1)
Ecuador	4	(1.1)
Nicaragua	3	(0.8)
Paraguay	2	(0.5)
Peru	2	(0.5)
**Other countries of birth**		
United States	43^¶^	(11.8)
Countries in Europe	2**	(0.5)
Not specified	1	(0.3)
**Phase of *Trypanosoma cruzi* infection**		
Acute infection	4	(1.1)
Chronic infection, not reactivated	326	(89.3)
Chronic infection, reactivated	35	(9.6)

* Data for four other patients were excluded from these intention-to-treat analyses: two patients who received benznidazole prophylaxis after laboratory accidents and two patients determined not to have Chagas disease after benznidazole was released.

^†^ Effective May 14, 2018, patient enrollment was discontinued.

^§^ Countries are listed in descending order by number of patients. Most of these patients likely became infected in Latin America, although not necessarily in their country of birth.

^¶^ Autochthonous, presumptive vectorborne transmission was considered the likely means by which at least 50% of the U.S.-born patients became infected, including one patient with an acute infection.

** One patient became infected in Mexico, and the other patient likely became infected in the United States via presumptive vectorborne transmission.

Overall, 319 patients (87.4%) had been born in Latin America, including a total of 261 (71.5%) from El Salvador (117), Mexico (77), or Bolivia (67) (Table). The shipping addresses of the physicians of record for the 365 patients were in 41 states and the District of Columbia; however, seven (16.7%) of the 42 jurisdictions accounted for 263 patients (72.1%): California (111 patients), Texas (40), New York (35), the District of Columbia (22), Massachusetts (21), Virginia (19), and Florida (15).

Only four patients (1.1%) had acute-phase infection (Table). All four were born and became infected in the United States, two via transplantation of solid organs from Central American immigrants, one via congenital transmission from a Bolivian immigrant (*8*), and one via presumptive vectorborne transmission. The other 361 patients (98.9%) had chronic-phase infection, 35 of whom were Latin American immigrants who developed reactivated infection after becoming immunosuppressed in the context of solid organ transplantation (29 patients), infection with human immunodeficiency virus (five), or chemotherapy (one).

## Conclusion

The majority of patients for whom CDC released benznidazole under the IND were chronically infected adults who were born and became infected in Latin America. Only two patients were aged 2–12 years, the group for which FDA has approved the use of benznidazole. However, FDA-approved drugs can be used for nonapproved indications (i.e., “off label”), in accordance with the practice of medicine (*7*). FDA’s approval of benznidazole and the commercial availability of the approved product in the United States likely will increase awareness of Chagas disease and facilitate access to therapy.

CDC will continue to provide reference diagnostic testing for *T. cruzi* infection (https://www.cdc.gov/dpdx) and teleconsultative services regarding Chagas disease. Health care providers and U.S. health departments with questions about Chagas disease may contact CDC Parasitic Diseases Branch Inquiries by telephone (404-718-4745) or e-mail (parasites@cdc.gov). The CDC Drug Service may be contacted by telephone (404-639-3670) or e-mail (drugservice@cdc.gov). Physician requests for benznidazole, which is now commercially available in the United States, should be directed to Exeltis at http://www.benznidazoletablets.com/en/ (telephone: 877-303-7181; e-mail: FastAccess@exeltis.com). Additional information about Chagas disease is available on CDC's website at https://www.cdc.gov/parasites/chagas.

**Summary**

**What is already known about this topic?**

Benznidazole is used to treat Chagas disease, a potentially life-threatening parasitic disease. In October 2011, a CDC-sponsored Investigational New Drug (IND) treatment protocol went into effect to ensure benznidazole availability for eligible U.S. patients.

**What is added by this report?**

Among 365 patients for whom CDC released benznidazole under the IND, 362 (99%) were aged ≥13 years, 361 (99%) had chronic-phase infection, and 319 (87%) were Latin American immigrants. CDC stopped enrolling patients in the IND program in May 2018, when a benznidazole product approved by the Food and Drug Administration in August 2017 became commercially available.

**What are the implications for public health practice?**

Physician requests for benznidazole should now be directed to the drug company Exeltis (http://www.benznidazoletablets.com/en/).

## Footnotes

* Exeltis is the U.S. regulatory representative for Chemo Research, SL.

† http://www.benznidazoletablets.com/en/.
